# Risk prediction of carbapenem-resistant *Pseudomonas aeruginosa* infection in children

**DOI:** 10.3389/fcimb.2026.1846657

**Published:** 2026-06-01

**Authors:** Lin Qin, Xiaoling Wei, Min Xue, Jie Chen, Xia Lin, Xiang Ma

**Affiliations:** 1 Department of Health Data Application and Management, Children’s Hospital Affiliated to Shandong University, Jinan, Shandong, China; 2 Shandong Provincial Clinical Research Center for Children’s Health and Disease, Children’s Hospital Affiliated to Shandong University, Jinan, Shandong, China; 3 Key Laboratory of Precision Diagnosis and Treatment of Pediatric Infectious Diseases, Jinan, Shandong, China; 4 Pediatric Intensive Care Unit, Children’s Hospital Affiliated to Shandong University, Jinan, Shandong, China; 5 Department of Respiratory Diseases, Children’s Hospital Affiliated to Shandong University, Jinan, Shandong, China; 6 Innovative Institute of Chinese Medicine and Pharmacy, Shandong University of Traditional Chinese Medicine, Jinan, Shandong, China

**Keywords:** carbapenem-resistant *Pseudomonas aeruginosa*, children, risk prediction model, Shap, XGBoost

## Abstract

**Objective:**

This study aimed to investigate the infection distribution, drug resistance, and risk factors associated with carbapenem-resistant *Pseudomonas aeruginosa* (PA) in children, and to construct a risk prediction model.

**Methods:**

Two retrospective cohorts were established based on the Children’s Hospital Affiliated to Shandong University. The derivation cohort included 1397 children with *Pseudomonas aeruginosa* infection hospitalized from January 2020 to December 2024, divided into the carbapenem-resistant PA (CRPA) group and the carbapenem-sensitive PA (CSPA) group based on antimicrobial susceptibility testing. LASSO regression was used for feature selection, and an XGBoost model was constructed to predict CRPA infection, with internal validation performed using a randomly split test set. Subsequently, an independent temporal validation cohort of 431 children (January 2025 to March 2026) was used to evaluate model generalizability. SHapley Additive exPlanations (SHAP) was employed to interpret variable importance.

**Results:**

Among the 1397 PA isolates, 1177 were CSPA and 220 were CRPA. Children in the CRPA group were younger, had longer hospital stays, and were more prevalent in the NICU and PICU than those in the CSPA group. Among CRPA isolates, 88.2% met multidrug-resistant criteria and 46.4% met extensively drug-resistant criteria, while maintaining high sensitivity to amikacin (95%). The XGBoost model, incorporating 10 key variables, achieved an AUC of 0.848 (95% CI, 0.783–0.912) on the internal test set and 0.715 (95% CI, 0.622–0.807) on the temporal validation cohort. SHAP interpretability analysis showed that length of hospital stay, CD8^+^ T cells, ventilator use, and pre-infection carbapenem exposure were important risk factors for CRPA infection.

**Conclusion:**

CRPA infection in children is closely associated with prolonged hospitalization, immune dysregulation, invasive procedures, and prior carbapenem exposure. The XGBoost prediction model demonstrated good discrimination in internal validation, and its performance remained stable on temporal validation, suggesting potential utility in identifying high-risk children for early clinical intervention.

## 1 Introduction

*Pseudomonas aeruginosa* (PA) is a prevalent opportunistic pathogen associated with hospital infections. Its inherent drug resistance and robust environmental adaptability bring severe challenges to clinical treatment. In recent years, the wide application of broad-spectrum antibiotics, such as carbapenems, has led to a significant increase in the global detection rate of carbapenem-resistant *P. aeruginosa* (CRPA) (Tenover et al., 2022). CRPA strains are usually characterized by multi-drug resistance (MDR) and even extensively drug resistance, resulting in limited treatment options, prolonged hospital stays, elevated medical costs and increased mortality, which poses a major threat to global public health (Antimicrobial Resistance Collaborators, 2022; Wangchinda et al., 2024; van Veen et al., 2025). The 2024 National Bacterial Resistance Monitoring Report showed that PA accounted for 11.3% of the isolated Gram-negative bacteria, second only to *Escherichia coli* (28.6% of Gram-negative bacteria) and *Klebsiella pneumoniae* (21.3% of Gram-negative bacteria) (System. A. R. S, 2025). The detection rate of CRPA was 16%. Among them, the detection rate of CRPA in Chinese newborns was 13.2%, whereas that in Chinese children was 6.2% (System. A. R. S, 2025). Children, especially newborns and critically ill children, are susceptible and at high risk of CRPA infection because of their underdeveloped immune systems, complex underlying conditions and frequent need to undergo various invasive procedures (Rodríguez et al., 2024). Therefore, this study collected clinical data of children, used LASSO regression for feature selection, constructed a risk prediction model for CRPA resistance based on XGBoost, and utilized the SHAP method for interpretable analysis, aiming to provide a reference for the early prevention and prompt identification of CRPA infections in children.

## 2 Materials and methods

### 2.1 Research object

This study was performed using two retrospective cohorts from the Children’s Hospital Affiliated to Shandong University. The derivation cohort consisted of all children hospitalized from January 2020 to December 2024 with *P. aeruginosa* isolated by microbial culture (n = 1397), with which we trained and performed an internal validation of the prediction model. The temporal validation cohort consecutively included all children meeting the identical criteria from January 2025 to March 2026 (n = 431), with which we performed an independent temporal validation of the prediction model.

Pathogen-positive specimens including sputum, alveolar lavage fluid, catheter head, wound secretions, midstream urine, and peritoneal effusion. At least one positive culture result was required from the same or different specimens; if multiple positive results were obtained, the first positive specimen confirming clear infection was selected for analysis. This study was approved by the ethics committee of our hospital (SDFE-IRB/T-2026032).

#### 2.1.1 Inclusion criteria

1. Age 0–18 years;
2. At least one clinical specimen culture-positive for *P. aeruginosa*;
3. The positive culture episode met the clinical criteria for infection (see Section 2.2).

#### 2.1.2 Exclusion criteria

1. Episodes judged as colonization rather than infection according to the criteria in Section 2.2;
2. Patients with prior confirmed CRPA infection or colonization during the same hospitalization episode.

### 2.2 Definition of infection versus colonization

The distinction between infection and colonization was made according to the 2022 Chinese Consensus on the Diagnosis and Treatment of *Pseudomonas aeruginosa* Lower Respiratory Tract Infection, with additional reference to the CDC/NHSN surveillance frameworks (Chinese Thoracic Society et al., 2022; Horan et al., 2008). A culture-positive episode was classified as infection if it met all of the following criteria:

1. Clinical signs: new-onset fever (> 38.0 °C), new or worsening respiratory symptoms (cough, purulent sputum, dyspnea), or systemic signs of sepsis.
2. Laboratory evidence: elevated C-reactive protein (> 10 mg/L) or procalcitonin (> 0.5 ng/mL), or abnormal white blood cell count (> 15×10^9^/L or < 4×10^9^/L).
3. Treatment response: the treating physician initiated or escalated anti-pseudomonal antibiotic therapy, with documented clinical improvement thereafter.

For mechanically ventilated patients, an additional requirement was applied: oxygenation deterioration (increase in FiO_2_ ≥ 0.2 or PEEP ≥ 2 cmH_2_O) accompanied by purulent tracheal secretions, within 72 hours before specimen collection.

Episodes not meeting all criteria were considered probable colonization and excluded.

### 2.3 Data collection

The prediction time point was defined as the collection date of the index specimen that first yielded *P. aeruginosa*. Data were obtained from the Hospital Health Medical Big Data Research and Innovation Platform, including the following:

1. Demographic and basic clinical characteristics: age, gender, specimen type, admission department, and length of hospital stay (calculated from admission to the index specimen collection). HIV serostatus was ascertained from pre-infection laboratory records. Among 971 children with available results, all were seronegative. HIV status was therefore not included as a candidate predictor.
2. Laboratory indicators: blood routine and inflammatory markers, including white blood cell count, neutrophil count and its ratio, lymphocyte count and its ratio, red blood cell count, hemoglobin, platelet count, C-reactive protein, procalcitonin and erythrocyte sedimentation rate; coagulation and immune function-related indicators, including fibrinogen, D-dimer, international normalized ratio, immunoglobulin A, immunoglobulin G, immunoglobulin M, relative count of helper/inducible T cells, relative count of inhibitory/cytotoxic T cells and CD4^+^/CD8^+^ ratio; cytokine detection included interleukin 2, interleukin 4, interleukin 6, interleukin 10, tumor necrosis factor and interferon γ. All laboratory values were the most recent results prior to the index specimen collection.
3. Previous treatment and drug exposure history: glucocorticoid administration, use of antifungal drugs, pre-infection carbapenem exposure, use of antimicrobial drugs and immunoglobulin therapy. Pre-infection carbapenem exposure was defined as any prescription of imipenem, meropenem, or other carbapenems occurring after admission but before the index specimen collection date, ascertained from electronic medication orders.
4. Invasive operation history: urinary catheter, central venous catheterization, mechanical ventilation, indwelling of gastric and drainage tubes, invasive puncture and flexible endoscopy. All procedures were recorded if they occurred between admission and the index specimen collection date.

### 2.4 Bacterial detection method and drug sensitivity test results

The VITEK 2-compact automatic microbial analysis system was used in the microbiological laboratory. The drug sensitivity results were interpreted according to the drug sensitivity test methods recommended by the American Society for Clinical and Laboratory Standards Institute (CLSI), including resistant, intermediate and susceptible. The quality control strains were *E. coli* ATCC 25922 and PA ATCC 27853. Susceptibility tests included Amikacin (2-64 µg/mL), Aztreonam (1-64 µg/mL), Cefepime (0.12-232 µg/mL), Cefoperazone/Sulbactam (8/4-64/32 µg/mL), Ceftazidime (0.12-64 µg/mL), Ciprofloxacin (0.25-4 µg/mL), Imipenem (0.25-16 µg/mL), Levofloxacin (0.12-8 µg/mL), Meropenem (0.25-16 µg/mL), Piperacillin/Tazobactam (4/4-128/4 µg/mL), Ticarcillin/Clavulanate (8/2-128/2 µg/mL), and Tobramycin (1-16 µg/mL). Confirmatory testing by disk diffusion was used for Aztreonam.

According to the results of drug sensitivity test, the strains were divided into the carbapenem-resistant PA (CRPA) group (resistant to one of the carbapenem antibiotics of meropenem or imipenem) and the carbapenem-sensitive PA (CSPA) group.

Multidrug-resistant (MDR) and extensively drug-resistant (XDR) phenotypes were defined according to the international expert consensus proposed by Magiorakos et al (Magiorakos et al., 2012). MDR was defined as non-susceptibility to at least one agent in three or more antimicrobial categories. XDR was defined as non-susceptibility to at least one agent in all but two or fewer categories. Based on the consensus classification for *Pseudomonas aeruginosa* and the results of antimicrobial susceptibility testing, 12 agents were grouped into six categories: (1) aminoglycosides (tobramycin, amikacin); (2) carbapenems (imipenem, meropenem); (3) cephalosporins (ceftazidime, cefepime, cefoperazone/sulbactam); (4) monobactams (aztreonam); (5) fluoroquinolones (ciprofloxacin, levofloxacin); and (6) penicillins plus *β*-lactamase inhibitors (piperacillin/tazobactam, ticarcillin-clavulanate). Each CRPA isolate was evaluated for the number of categories in which at least one agent was non-susceptible.

### 2.5 Statistical analysis

R 4.5.1 statistical software was used for data analysis. Normally distributed continuous variables were expressed as mean ± standard deviation ( $\bar{x}$ ± s), and the independent sample t test was used for comparison between groups. Non-normally distributed continuous variables were expressed as median (interquartile range) [M(Q1, Q3)], and the Mann–Whitney U test was used for comparison between groups. Categorical variables were described by frequency and composition ratio [n(%)]. The groups were compared by the *χ*^2^ test, corrected *χ*^2^ test or Fisher’s exact test. *P* < 0.05 indicated that the difference was statistically significant.

Variables with a missing rate of more than 50% were eliminated, and the multiple imputation method (generating five datasets) was used to address the remaining variables with missing values. Missing data proportions for all candidate predictors are summarized in **Supplementary Table 1**. Least Absolute Shrinkage and Selection Operator (LASSO) regression was used for feature selection, and the optimal penalty parameter (*λ*.*min*) was determined by tenfold cross-validation. To evaluate the robustness of feature selection to the missing data approach, two sensitivity analyses were conducted. First, the variables selected by LASSO in each of the five imputed datasets were recorded, and only those consistently selected across all five imputations were retained in the final model. Second, LASSO was repeated on the complete case subset (n = 304), and the resulting variable set was compared with that from the multiply imputed analysis.

The derivation cohort was randomly divided into the training set (n=1117) and the test set (n=280) at an 8:2 ratio by stratified sampling. The CRPA prediction model was constructed by the XGBoost algorithm. The hyperparameters of the model were optimized by fivefold cross-validation and random grid search. The final model was then evaluated on both the internal test set and the independent temporal validation cohort (n = 431, January 2025 to March 2026). The performance of the model was evaluated using sensitivity, specificity, positive predictive value, negative predictive value, area under the receiver operating characteristic curve (AUC–ROC), area under the precision–recall curve (AUPR), and brier score. To obtain a stable estimate of performance indicators, we calculated the 95% confidence interval (CI) of AUC–ROC via the DeLong method, while the 95% CI for other indicators was obtained by bootstrap resampling (1000 iterations). The SHapley Additive exPlanations (SHAP) method was used for interpretability analysis, and the importance and influence mechanisms of each factor were visually expressed.

### 2.6 Clinical utility assessment

To evaluate the clinical applicability of the model beyond ROC-AUC, two decision thresholds were identified on the test set. The screening threshold was determined by maximizing Youden’s index, prioritizing high sensitivity and negative predictive value to enable safe antibiotic de-escalation (i.e., replacing empiric broad-spectrum antibiotics with narrower-spectrum alternatives when the model rules out resistant infection). The targeted treatment threshold was selected to achieve a sufficiently high positive predictive value, thereby providing clinicians with a basis for making clinical decisions regarding targeted therapy against carbapenem-resistant *Pseudomonas aeruginosa*. Performance metrics at both thresholds were reported with 95% confidence intervals estimated via stratified bootstrap resampling (1,000 iterations).

### 2.7 Comparison with logistic regression

A logistic regression model was constructed using the same predictor variables identified by LASSO. The model was fitted on the same training set and evaluated on the same test set using identical performance metrics and bootstrap procedures. Odds ratios with 95% confidence intervals were calculated to complement the SHAP-based interpretability of the XGBoost model.

## 3 Results

### 3.1 Baseline characteristics and clinical distribution of CRPA and CSPA

A total of 1397 children with detected PA were included in the study, comprising 1177 cases in the CSPA group and 220 cases in the CRPA group. The baseline characteristics, department distribution and specimen sources of the two groups of patients are shown in **Table 1**, **Table 2** and **Table 3**, respectively.

**Table 1:** Clinical characteristics of CRPA and CSPA.

Variable	CSPA N=1177	CRPA N=220	Z/t/χ^2^	*P* value
Age(years)	1.46 (0.5, 5.24)	0.78 (0.2, 5.92)	2.609	0.009
Gender(girl)	404 (34.3%)	84 (38.2%)	1.05	0.306
Length of hospital stay	9 (7, 16)	26 (11, 52.25)	-11.395	<0.001
Neutrophil percentage	59.95 (37.8, 79.6)	67.8 (49.85, 79.6)	-2.98	0.003
Red blood cell count	4.52 (4.13, 4.85)	4.3 (3.78, 4.72)	4.542	<0.001
White blood cell count	12.57 (9.37, 16.8)	12.91 (9.77, 18.12)	-1.123	0.262
Neutrophil count	6.52 (3.27, 11.94)	8.01 (5.16, 12.49)	-2.933	0.003
Hemoglobin	123 (112, 133)	119 (108, 132.5)	1.497	0.134
Platelet count	434.5 (339.25, 539)	405 (296, 506.5)	2.799	0.005
Lymphocyte percentage	29.2 (13.1, 50.95)	22.4 (13.15, 35.75)	3.626	<0.001
Lymphocyte count	2.68 (1.47, 4.44)	2.21 (1.52, 3.38)	2.914	0.004
Fibrinogen	3.14 (2.24, 4.41)	2.59 (1.84, 3.59)	4.477	<0.001
Procalcitonin	0.1 (0.06, 0.3)	0.17 (0.08, 1.14)	-4.773	<0.001
Erythrocyte sedimentation rate	10 (3, 24)	13 (5, 31)	-2.01	0.043
C-reactive protein	7.97 (3.11, 55.62)	9.07 (3.11, 46.76)	0.438	0.661
D-dimer	0.87 (0.39, 2.96)	0.73 (0.44, 2.07)	0.269	0.788
International standardized ratio	1.11 (1.01, 1.23)	1.12 (0.99, 1.28)	-0.61	0.542
CD4^+^/CD8^+^ ratio	1.69 (1.17, 2.32)	1.29 (0.84, 2.02)	3.582	<0.001
CD4^+^ T cell percentage	34.96 ± 10.91	33.9 ± 12.7	0.898	0.370
CD8^+^ T cell percentage	21.06 (16.44, 26.16)	25.44 (18.36, 34.32)	-4.078	<0.001
Interleukin-2	1.56 (1.02, 3.06)	1.59 (1.1, 3.16)	-0.619	0.537
Interleukin-4	1.43 (1.03, 2.06)	1.81 (1.19, 2.24)	-1.657	0.098
Interleukin-6	41.76 (10.62, 201.8)	13.77 (7.58, 61.33)	2.477	0.013
Interleukin-10	7.64 (3.76, 23.93)	6.22 (3.52, 13.34)	0.895	0.371
Tumor necrosis factor	1.58 (1.15, 2.43)	1.77 (1.34, 2.82)	-1.69	0.091
Interferon-γ	1.83 (1.22, 2.96)	2.2 (1.34, 2.98)	-0.905	0.366
Immunoglobulin A	0.36 (0.16, 0.74)	0.39 (0.14, 1.25)	-1.011	0.312
Immunoglobulin G	6 (4.22, 8.79)	7.78 (5.2, 10.85)	-3.553	<0.001
Immunoglobulin M	0.78 (0.52, 1.16)	0.8 (0.4, 1.31)	0.05	0.960
Use of intravenous immunoglobulin	246 (20.9%)	74 (33.6%)	16.311	<0.001
Use of glucocorticoids	625 (53.1%)	137 (62.3%)	5.924	0.015
Use of antifungal agents	99 (8.4%)	61 (27.7%)	66.302	<0.001
Prior carbapenem exposure	282 (24%)	123 (55.9%)	90.365	<0.001
Use of antibacterial drugs	1141 (96.9%)	213 (96.8%)	0.01	0.920
Urinary catheterization	419 (35.6%)	71 (32.3%)	0.76	0.383
Mechanical ventilation	301 (25.6%)	151 (68.6%)	155.086	<0.001
Central venous catheterization	180 (15.3%)	79 (35.9%)	50.807	<0.001
Indwelling gastric tube or drainage tube	645 (54.8%)	182 (82.7%)	58.699	<0.001
Invasive puncture	121 (10.3%)	47 (21.4%)	20.486	<0.001
Flexible endoscopy	176 (15%)	67 (30.5%)	29.927	<0.001

**Table 2:** Department distribution of CRPA and CSPA.

Department	CSPA N = 1,177^1^	CRPA N = 220^1^	*p*-value^2^
			<0.001
Pediatric Intensive Care Unit	121 (10.3%)	57 (25.9%)	
Respiratory Intervention Department	138 (11.7%)	43 (19.5%)	
Respiratory Department	285 (24.2%)	13 (5.9%)	
Rehabilitation Department	15 (1.3%)	14 (6.4%)	
General Surgery	207 (17.6%)	9 (4.1%)	
Neonatal Intensive Care Unit	105 (8.9%)	59 (26.8%)	
Other	306 (26.0%)	25(11.4%)	

1 n (%)

2 Pearson’s Chi-squared test.

**Table 3:** Specimen type of CRPA and CSPA.

Specimen type	CSPA N = 1,177^1^	CRPA N = 220^1^	*p*-value^2^
			<0.001
catheter head	19 (1.6%)	6 (2.7%)	
bronchoalveolar lavage fluid	60 (5.1%)	21 (9.5%)	
pus	278 (23.6%)	11 (5.0%)	
sputum	724 (61.5%)	159 (72.3%)	
midstream urine	52 (4.4%)	11 (5.0%)	
other	44(3.7%)	12 (5.5%)	

1 n (%).

2 Pearson’s Chi-squared test.

**Table 1** shows the demographic and clinical characteristics of PA-infected patients. Compared with the CSPA group, the CRPA group was younger by 0.78 (0.2, 5.92) years, experienced a significantly prolonged hospital stay of 26 (11, 52.25) days and exhibited elevated levels of inflammatory markers PCT and NEU (*P* < 0.01). In terms of immune function, the LYM ratio and CD4^+^/CD8^+^ ratio of patients in the CRPA group were significantly lower than those in the CSPA group, whereas the proportion of CD8^+^ T cells was higher in the CRPA group than in the CSPA group (*P* < 0.001). In addition, patients in the CRPA group had higher IgG levels than those in the CSPA group. In terms of treatment history and invasive operations, the proportion of patients with previous carbapenem antibiotic exposure, immunoglobulin use, glucocorticoids use, antifungal treatment, mechanical ventilation, central venous catheterization and other invasive operations in the CRPA group was significantly higher than that in the CSPA group (*P* < 0.05).

Department distribution analysis (**Table 2**) showed a significant difference in department distribution between the two groups (*P* < 0.001). Children with CRPA infection were mainly from the Neonatal Intensive Care Unit (NICU, 26.8%), Pediatric Intensive Care Unit (PICU, 25.9%) and Respiratory Intervention Department (19.5%). The distribution of specimen types (**Table 3**) showed a significant difference between the two groups (*P* < 0.001). Sputum was the main source of specimens in the two groups, with the CRPA group accounting for 72.3%.

### 3.2 Detection of bacterial resistance

The study compared the resistance rates of the CRPA group and the CSPA group to 12 commonly used antibiotics. The results of the drug sensitivity test (**Table 4**) showed that the resistance rate of the CRPA group was higher than that of the CSPA group. The resistance rates of the CRPA group to imipenem and meropenem were 87.7% and 71.8%, respectively. The resistance rate of the CRPA group to ticarcillin/clavulanate potassium was 64.6%. Among the 220 CRPA isolates, 194 (88.2%) met the MDR criteria, and 102 (46.4%) were further classified as XDR. The two groups of bacteria isolated were highly sensitive to amikacin. The sensitivity rates of the CRPA group and the CSPA group were 95% and 98.6%, respectively.

**Table 4:** Antibiotic susceptibility test results of CRPA and CSPA.

	CRPA			CSPA				
Antibiotics	Sensitive	Intermediary	Resistant	Sensitive	Intermediary	Resistant	Statistic	*P* value
Imipenem	4 (1.8%)	23 (10.5%)	193 (87.7%)	1177 (100%)	0 (0%)	0 (0%)	-^1^	<0.001
Piperacillin/tazobactam	136 (61.8%)	28 (12.7%)	56 (25.5%)	1069 (90.8%)	28 (2.4%)	80 (6.8%)	133.875	<0.001
Ceftazidime	146 (66.4%)	23 (10.5%)	51 (23.2%)	1074 (91.2%)	29 (2.5%)	74 (6.3%)	104.066	<0.001
Cefepime	138 (62.7%)	29 (13.2%)	53 (24.1%)	1091 (92.7%)	31 (2.6%)	55 (4.7%)	157.339	<0.001
Cefoperazone/Sulbactam	89 (50%)	49 (27.5%)	40 (22.5%)	973 (96.1%)	33 (3.3%)	7 (0.7%)	347.542	<0.001
Tobramycin	196 (89.1%)	7 (3.2%)	17 (7.7%)	1157 (98.3%)	6 (0.5%)	14 (1.2%)	–	<0.001
Levofloxacin	172 (78.2%)	29 (13.2%)	19 (8.6%)	1145 (97.3%)	14 (1.2%)	18 (1.5%)	129.125	<0.001
Ticarcillin - Clavulanate	18 (9.1%)	52 (26.3%)	128 (64.6%)	367 (33.2%)	621 (56.2%)	117 (10.6%)	323.168	<0.001
Aztreonam	72 (32.7%)	49 (22.3%)	99 (45%)	982 (83.9%)	63 (5.4%)	125 (10.7%)	264.896	<0.001
Ciprofloxacin	62 (28.2%)	19 (8.6%)	139 (63.2%)	1177 (100%)	0 (0%)	0 (0%)	–	<0.001
Meropenem	36 (16.4%)	26 (11.8%)	158 (71.8%)	1177 (100%)	0 (0%)	0 (0%)	–	<0.001
Amikacin	209 (95%)	4 (1.8%)	7 (3.2%)	1160 (98.6%)	10 (0.9%)	6 (0.5%)	–	0.002

1 Fisher’s exact test.

### 3.3 LASSO regression

**Figure 1:**
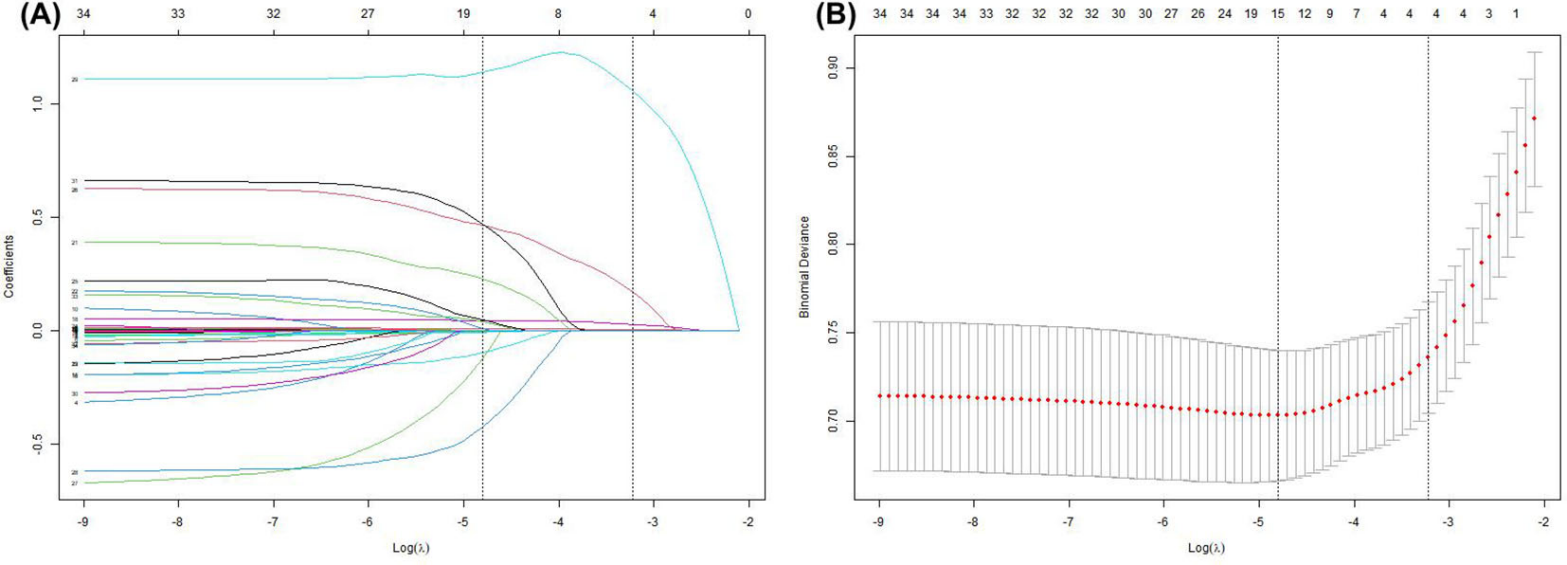
LASSO regression results for the first imputed dataset. **(A)** coefficient path diagram, **(B)** cross-validation diagram. The two dotted vertical lines indicate *λ*. *min*(left) and *λ*. *1se* (right). Numbers at the top denote the number of non-zero variables at each penalty level.

LASSO regression was used to select features from the above 34 variables to address multicollinearity among variables. By introducing the L1 penalty term, the coefficients of non-important variables were reduced to zero, achieving feature selection. For the five data sets generated by multiple imputation, LASSO regression was fitted independently, and the optimal regularization parameters were determined by tenfold cross-validation. **Figure 1** shows the LASSO variable selection process for the first imputed dataset. The left dotted line represents *λ*. *min*, and the right dotted line denotes *λ*. *1se*. Using *λ*. *min* as the standard, the selection frequency of variables in each data set was counted, and the variables selected in all five data sets were retained as stable predictors. The variable selection frequency is shown in **Supplementary Table 2**. The final screening variables comprised CD8^+^ T cells, D-dimer, fibrinogen, length of hospital stay, LYM ratio, gender, urinary catheter, previous carbapenem antibiotic exposure, mechanical ventilation, gastric tube and drainage tube indwelling.

On the complete case subset (n = 304), LASSO selected 10 variables at *λ*. *min* (**Supplementary Table 2**), of which six overlapped with the multiply imputed analysis: CD8^+^ T cells, D-dimer, previous carbapenem antibiotic exposure, mechanical ventilation, gastric tube and drainage tube indwelling, and gender. The divergence in the remaining variables underscores the necessity of multiple imputation for stable feature selection in this dataset.

### 3.4 Construction of XGBoost prediction model

**Figure 2:**
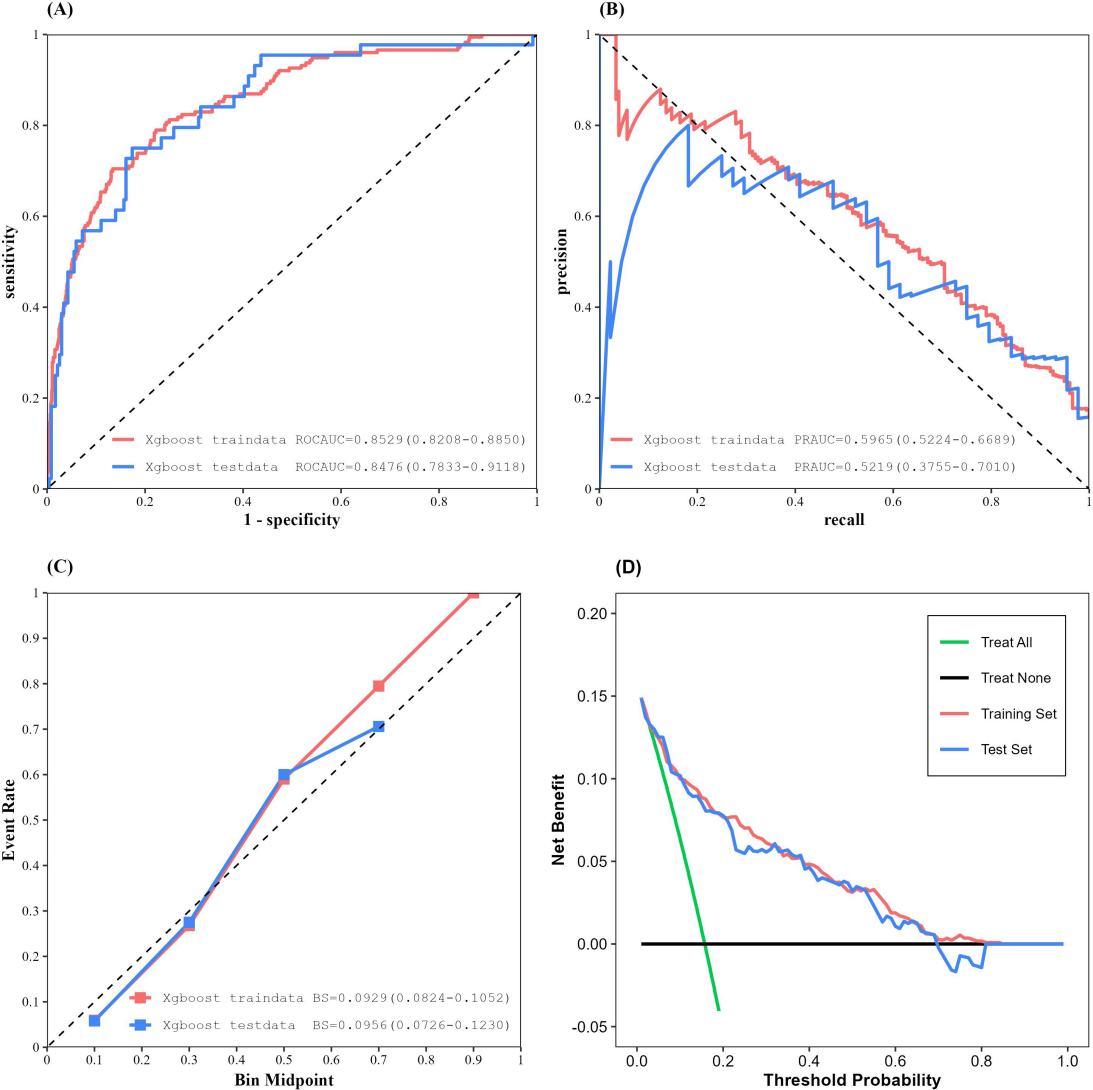
Model performance evaluation. **(A)** ROC curves, **(B)** precision-recall curves, **(C)** calibration curves, and **(D)** decision curve analysis for the training (red) and test (blue) sets.

The variables derived from feature selection were incorporated into the XGBoost classification prediction model. In the model training stage, fivefold cross-validation combined with a random grid search strategy was used to systematically optimize the key hyperparameters including tree depth, learning rate and feature sampling number. The comprehensive performance evaluation results of the prediction model on the derivation cohort are shown in **Figure 2**. To obtain stable estimates, the 95% confidence interval (CI) of AUC–ROC was calculated via the DeLong method, while the 95% CI for all other indicators was obtained by stratified bootstrap resampling (1,000 iterations). The area under the receiver operating characteristic curve (ROC–AUC) of the training set was 0.853 (95% CI: 0.821–0.885), and that for the test set was 0.848 (95% CI: 0.783–0.912). On the independent temporal validation cohort (n = 431, January 2025 to March 2026), the model achieved an ROC–AUC of 0.715 (95% CI: 0.622–0.807). The detailed information of the model performance indicators is shown in **Table 5**.

**Table 5:** The performance metrics of the prediction model.

Metrics	Training set	Test set	Temporal validation
ROCAUC	0.853 (0.821, 0.885)	0.848 (0.783, 0.912)	0.715 (0.622, 0.807)
PRAUC	0.597 (0.522, 0.669)	0.522 (0.376, 0.701)	0.240 (0.153, 0.373)
Sensitivity	0.705 (0.632, 0.770)	0.636 (0.478, 0.773)	0.476 (0.337, 0.625)
Specificity	0.866 (0.846, 0.887)	0.843 (0.792, 0.890)	0.889 (0.859, 0.920)
Precision (PPV)	0.496 (0.438, 0.554)	0.431 (0.342, 0.531)	0.317 (0.218, 0.428)
NPV	0.940 (0.923, 0.955)	0.926 (0.897, 0.952)	0.940 (0.917, 0.962)
Brier Score	0.093 (0.082, 0.105)	0.096 (0.073, 0.123)	0.083 (0.065, 0.102)

NPV, negative predictive value; PPV, positive predictive value.

### 3.5 Clinically meaningful decision thresholds

**Figure 3:**
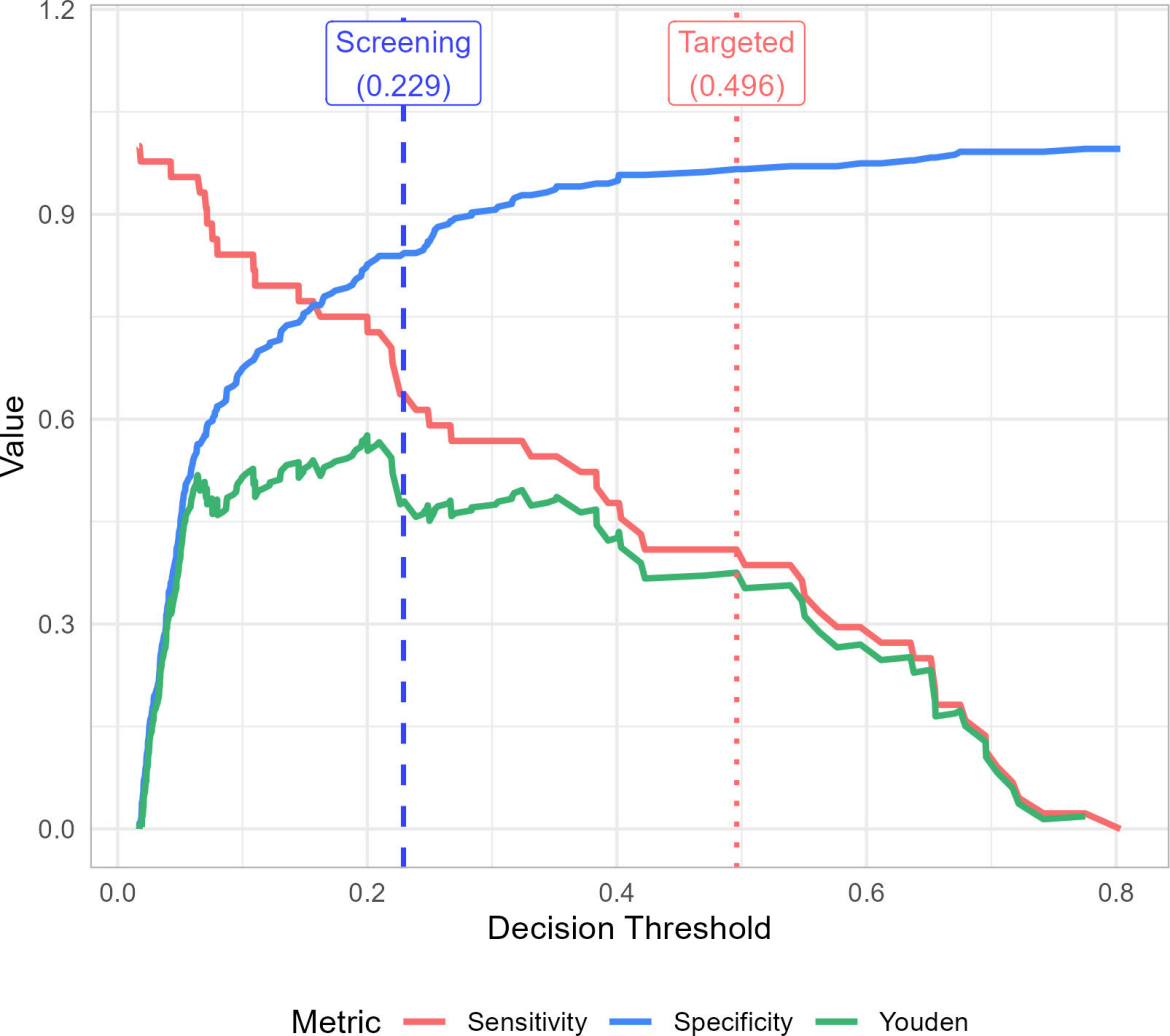
Performance metrics across different decision thresholds on the test set. The blue dashed line indicates the Youden-optimal screening threshold (0.229), which balances sensitivity and specificity and may help support de-escalation from broad-spectrum to narrow-spectrum antibiotics. The red dotted line indicates the targeted treatment threshold (0.496), where specificity and positive predictive value are substantially higher.

**Figure 3** illustrates the trade-off between sensitivity and specificity across the full range of decision thresholds on the test set. The Youden-optimal screening threshold (0.229) and the targeted treatment threshold (0.496) are indicated.

At the screening threshold of 0.229, the model achieved a sensitivity of 0.636 (95% CI: 0.500–0.750), specificity of 0.843 (95% CI: 0.795–0.892), PPV of 0.431 (95% CI: 0.342–0.538), and NPV of 0.926 (95% CI: 0.899–0.949) (**Table 6**). The high NPV suggests that, when the model assigns a low-risk label, 92.6% of these children truly do not harbor CRPA, which may assist clinicians in identifying patients who could be candidates for antibiotic de-escalation.

**Table 6:** Performance of the XGBoost model at two clinically meaningful decision thresholds on the test set.

Metric	Screening (Youden)	Targeted treatment
Threshold	0.229	0.496
Sensitivity (95% CI)	0.636 (0.478, 0.773)	0.409 (0.250, 0.523)
Specificity (95% CI)	0.843 (0.792, 0.890)	0.966 (0.941, 0.987)
PPV (95% CI)	0.431 (0.342, 0.531)	0.692 (0.515, 0.846)
NPV (95% CI)	0.926 (0.897, 0.952)	0.898 (0.873, 0.917)

To address the limited PPV at the screening threshold, a targeted treatment threshold of 0.496 was identified. At this threshold, the PPV improved to 0.692 (95% CI: 0.520–0.847), specificity reached 0.966 (95% CI: 0.941–0.987), while sensitivity decreased to 0.409 (95% CI: 0.239–0.523). When applied at this higher threshold, approximately 70% of children predicted as high-risk are confirmed to be true CRPA infection. This information may serve as a reference for clinicians when assessing the need for antibiotic therapy targeting carbapenem-resistant *Pseudomonas aeruginosa*, but should be interpreted together with other clinical and epidemiological factors. It should be emphasized that the model is intended as an adjunctive risk-stratification tool, rather than the sole basis for treatment decisions.

### 3.6 Interpretability analysis based on SHAP

**Figure 4:**
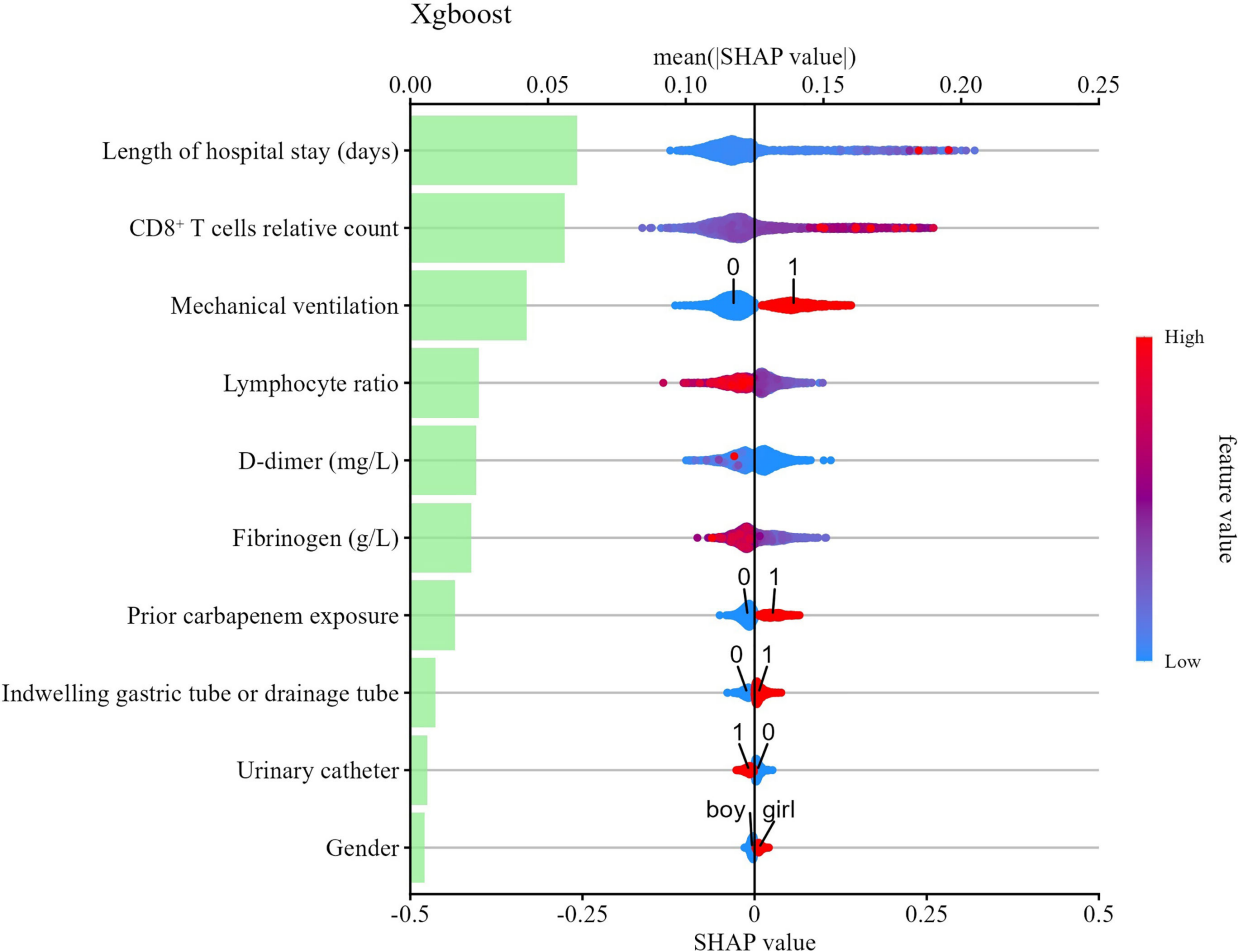
SHAP summary plot. Green bars represent mean absolute SHAP values, ranking feature importance. Colored points represent SHAP values for individual predictions: points to the right of zero push the prediction toward carbapenem-resistant *Pseudomonas aeruginosa* (CRPA), and points to the left push toward carbapenem-sensitive *Pseudomonas aeruginosa* (CSPA), with color indicating feature magnitude (red = high, blue = low).

The machine learning model demonstrated good predictive ability in clinical prediction research, but elucidating its black box functionality poses challenges. The SHAP method can effectively solve this problem. The SHAP summary plot (**Figure 4**) shows the global effect of each feature variable on the prediction results. Each point in the figure represents the feature SHAP value of a sample (a SHAP value greater than 0 indicates that the feature is positively correlated with the risk of drug resistance, and a SHAP value is less than 0 suggests the opposite trend). The color of the point represents the value of the feature (red signifies high value, whereas blue indicates low value), and the features are sorted from top to bottom according to the average degree of influence on the model output (i.e. the average SHAP absolute value). The analysis results showed that the importance of each characteristic variable was as follows: length of hospital stay, CD8^+^ T cell frequency, ventilator use, lymphocyte ratio, D-dimer, fibrinogen, previous carbapenem exposure before infection, use of a gastric tube and drainage tube during hospitalization, urinary catheter insertion within the past year, and patient gender. Simultaneously, prolonged hospital stay, high CD8^+^ T cell frequency, ventilator use and early-stage carbapenem exposure are important risk factors for the model to predict CRPA resistance.

Furthermore, we quantified the contribution of each feature to individual predictions of CRPA infection risk in children by analyzing local SHAP values (**Supplementary Figure 1**). **Supplementary Figure 1** presents a SHAP force plot for a single pediatric patient: prolonged hospital stay, mechanical ventilation, and prior carbapenem exposure were positive contributors that significantly increased the model’s predicted risk of CRPA infection, while CD8^+^ T cells and D-dimer were negative contributors that decreased the predicted risk.

### 3.7 Comparison with logistic regression

A logistic regression model was constructed using the same predictor variables identified by LASSO. On the internal test set, the logistic regression model achieved an AUC of 0.795 (95% CI, 0.729–0.860), sensitivity of 0.727, specificity of 0.766, and NPV of 0.938. Detailed performance metrics of the logistic regression model are provided in **Supplementary Table 3**.

The logistic regression model showed modestly lower discrimination than XGBoost (AUC 0.795 vs. 0.848) but slightly higher sensitivity and NPV, suggesting that it may be particularly suitable as a screening tool for ruling out low-risk patients. The odds ratios from the logistic regression model are presented in **Supplementary Table 4**. Mechanical ventilation, prior carbapenem exposure, CD8^+^ T cell count, and length of hospital stay were significant independent predictors of CRPA, consistent with their clinical relevance in the XGBoost model.

## 4 Discussion

This study was performed using two retrospective cohorts from the Children’s Hospital Affiliated to Shandong University. On the basis of the clinical data of 1397 children with PA infection in the derivation cohort, this study screened critical variables by LASSO regression and constructed an XGBoost model to predict the risk of CRPA infection. The model performed well in the training set and the test set (AUCs were 0.853 and 0.848, respectively). To further evaluate generalizability, we performed an independent temporal validation on an additional cohort of 431 children with PA infection hospitalized between January 2025 and March 2026. The model achieved an AUC of 0.715 (95% CI: 0.622–0.807) on this cohort. The moderate decline in discrimination relative to the internal test set likely reflects temporal shifts in resistance epidemiology and the lower CRPA prevalence in the temporal cohort (9.7% vs. 15.7%). The SHAP method was used to interpret the model, revealing the key risk factors affecting CRPA infection, providing a reference for the early identification and clinical intervention of CRPA infection in children.

We observed significant differences in basic clinical characteristics between the CRPA group and the CSPA group. The children in the CRPA group were younger and their hospital stay was significantly prolonged compared with those in the CSPA group. This suggests that young children, especially infants with underdeveloped immune systems, are more susceptible to CRPA infection (Dong et al., 2025). An extended hospital stay not only reflects a severe underlying disease but also increases the risk of exposure to opportunistic pathogens. In terms of department distribution, the children in the CRPA group were mainly from the PICU and NICU, while the CSPA group was mainly from the respiratory and general surgery departments. The PICU and NICU are specialized care units for critically ill children and those with underdeveloped immune systems in hospitals. Children in these departments often require invasive operations (such as mechanical ventilation and central venous catheterization), and the history of exposure to carbapenem antibiotics is prevalent. These factors collectively promote the emergence and dissemination of CRPA infection. The primary source of specimens in the CRPA and CSPA groups was sputum. Recent studies demonstrated that the predominant form of CRPA infection is respiratory tract infection (Jiang et al., 2025). Sputum specimens are easy to obtain in clinical settings, and ventilator tubes facilitate the development of CRPA biofilms, leading to recurrent infections and colonization that are challenging to remove. Therefore, the prevention of ventilator-associated pneumonia (VAP) is of vital importance. The 2024 updated expert consensus on VAP suggests the formation of a specialized infection control team to provide guidance and training on the prevention and treatment of VAP, as well as the rational use of antibiotics (Klompas et al., 2022; Rosenthal et al., 2025).

There were significant differences in inflammation and immune indexes between the CRPA group and the CSPA group. Procalcitonin levels and neutrophil counts were significantly higher in the CRPA group than in the CSPA group, which is consistent with previous studies (Peng et al., 2024). The IL-6 level in the CRPA group was lower than that in the CSPA group. This finding is consistent with a recent study showing that multidrug-resistant *Pseudomonas aeruginosa* strains, particularly those carrying the exoU gene, can induce a distinct cytokine profile characterized by decreased IL-6 and increased IL-1*β* expression (Badillo-Larios et al., 2024). These observations suggest that the lower IL-6 levels in CRPA infection may reflect strain-specific immune modulation rather than a milder infection severity. In terms of adaptive immune indicators, the IgG level was higher in the CRPA group than in the CSPA group. This observation aligns with findings in carbapenem-resistant *Klebsiella pneumoniae* (CRKP) infections, where resistant strains were shown to induce significantly higher titers of specific IgG antibodies compared to susceptible strains (Wang et al., 2022). These differences in immune indicators suggest that the basic immune status of children may be an important factor influencing susceptibility to CRPA infection.

The results on drug resistance showed that the CRPA group was not only generally resistant to carbapenems (such as imipenem and meropenem) but also showed a high resistance rate to frequently utilized antibiotics (such as monocyclic *β*-lactams and fluoroquinolones), along with multiple drug resistance. In this cohort, 88.2% of CRPA isolates were MDR and 46.4% were XDR. This high proportion of multidrug resistance considerably limits the available therapeutic options, especially for XDR strains. The extensive resistance to agents such as cephalosporins and piperacillin/tazobactam considerably narrows the choice of effective empiric therapy, which is particularly problematic in pediatric patients for whom antibiotic options are already limited by age and safety considerations. This means that while awaiting susceptibility results, the empirically chosen antibiotic is often ineffective against the resistant strain. The factors responsible for the MDR of CRPA include loss or down-regulation of outer membrane porin OprD; over-expression of active efflux pumps, such as MexAB-OprM, expels a variety of structurally diverse antibiotics from the bacteria; and carbapenemase genes, such as blaKPC and blaVIM, were obtained by mobile genetic elements such as plasmids, which enhanced the MDR of the strains (Yoon and Jeong, 2021; Lorusso et al., 2022; Falcone et al., 2024). The two groups of bacteria isolated were sensitive to amikacin. The sensitivity rates of the CRPA group and the CSPA group were 95% and 98.6%, respectively. The preserved susceptibility to amikacin in both groups supports its important role in combination therapy for pediatric CRPA infections, particularly for XDR strains with limited treatment options. Kon et al. conducted genome-wide sequencing and analysis of CRPA bloodstream isolates from multiple medical centers in Israel, revealing that under MDR to carbapenems and new *β*-lactam/enzyme inhibitor combinations, the vast majority of isolates (90.6%) are sensitive to amikacin (Kon et al., 2024). Therefore, in the clinical treatment of CRPA infection, we can refer to the local drug sensitivity monitoring data and adopt combination therapy utilizing amikacin.

The results of SHAP interpretability analysis showed that length of hospital stay, mechanical ventilation, immunological status (such as CD8^+^ T cell level) and previous medical intervention (such as pre-infection carbapenem exposure) were important risk factors for CRPA infection. Consistent with previous studies, the length of hospital stay is positively correlated with the increased risk of CRPA infection (Nabarro et al., 2017; Huang et al., 2023; Xu et al., 2025). Prolonged hospitalization reflects the severity of the child’s underlying disease, which often requires invasive procedures that damage the immune system and skin mucosal barrier (Shi et al., 2024). In addition, prolonged stay in the PICU or NICU directly increases the risk of exposure to and acquisition of drug-resistant pathogens. However, it should be noted that length of hospital stay may partially reflect worsening clinical status before infection, rather than purely baseline risk at admission. The model is therefore intended as a risk assessment tool at the time of culture collection, not as a prediction at hospital admission. Mechanical ventilation significantly increases the risk of CRPA infection, consistent with previous reports (Harte et al., 2021; Shi et al., 2024). Tracheal intubation injures the airway mucosal barrier and provides a direct pathway for pathogen invasion. In addition, the ventilator tube, particularly its moist inner surface, is susceptible to biofilm formation by pathogens, serving as a source of chronic infection. An elevated CD8^+^ T cell count was associated with an increased risk of CRPA infection (corresponding to the high CD8^+^ T cell count on the right side of the SHAP summary plot). This finding contrasts with a previous study on *Pseudomonas aeruginosa* infection, in which CD8^+^ T cell levels were reported to be decreased in PA-infected patients compared with non-PA-infected patients (Zhao et al., 2024). This discrepancy may be related to differences in the study populations or the distinct immune interactions induced by carbapenem-resistant strains. Pre-infection exposure to carbapenems significantly increases the risk of CRPA resistance, which is consistent with previous studies (Li et al., 2022; Shi et al., 2024; Guan et al., 2025). The administration of carbapenems during the early stage of infection leads to a decrease in pathogenic bacteria susceptible to antimicrobial agents, whereas the proliferation of resistant pathogenic bacteria increases the risk of CRPA infection. Reducing unnecessary antibiotic exposure can diminish the emergence of drug-resistant bacterial strains. The hospital’s clinical microbiology laboratory may regularly provide epidemiological bulletins to clinicians, which is crucial for effective empirical therapy, especially in patients at risk of VAP. Access to localized infection data enables clinicians to make informed decisions, formulate personalized treatment plans, and reduce incorrect antibiotic use.

The logistic regression model, as a simpler and inherently interpretable alternative, showed comparable sensitivity and NPV, making it a suitable screening tool for ruling out low-risk patients. However, the XGBoost model maintained clinical utility that the simpler model does not provide. Specifically, XGBoost achieved a higher PPV, which becomes crucial at the targeted treatment threshold, and its tree-based structure captures non-linear effects of certain predictors that were not significant in the logistic regression.

## 5 Limitation

This study is a single-center retrospective analysis, which may be subject to selection and information bias. Although the model was temporally validated on an independent cohort, external validation across multiple centers is still needed to confirm its generalizability to different clinical settings and patient populations. Some laboratory indicators are missing, and the stability of the model may still be affected by extensive imputation. Furthermore, despite the use of the clinical infection criteria, distinguishing lower respiratory tract infection from colonization remains challenging in mechanically ventilated children in PICU and NICU settings, and some degree of residual misclassification cannot be excluded. Vaccination history within 30 days prior to specimen collection was not systematically documented and therefore could not be evaluated; recent immunization may transiently elevate certain inflammatory markers, which is an unmeasured potential confounder. Future multi-center, prospective and large-sample studies are necessary to thoroughly elucidate the epidemiological characteristics and risk factors associated with CRPA infection in children.

## 6 Conclusion

CRPA infection in children is closely associated with prolonged hospital stay, immune dysregulation, invasive procedures, and prior carbapenem exposure. Based on these factors, the XGBoost-SHAP model can predict the risk of CRPA infection in children. The XGBoost model based on clinical data showed good discriminative ability in predicting CRPA infection in children. This work may serve as a reference for the early identification of high-risk children and the optimization of antimicrobial drug use strategies in pediatric clinics.

## Data Availability

The data analyzed in this study is subject to the following licenses/restrictions: Due to patient privacy regulations and ethical restrictions imposed by the Jinan Children’s Hospital Research Ethics Committee, the datasets analyzed in this study are not publicly available. Requests to access these datasets should be directed to Xiang Ma, maxiang0176@163.com.
