# Supplementary material for: Risk prediction of carbapenem-resistant Pseudomonas aeruginosa infection in children
Source: Front Cell Infect Microbiol. 2026 Jun 1;16:1846657. doi: 10.3389/fcimb.2026.1846657 (PMC13265335; doi:10.3389/fcimb.2026.1846657)
Supplement: Supplementary file 1 [file Table1.docx]

Supplementary Material

# Supplementary Tables

**Supplementary Table S1 : Missing data summary for all candidate predictors**

| **Variable** | **Missing_n** | **Missing_pct** | **Variable** | **Missing_n** | **Missing_pct** |
| --- | --- | --- | --- | --- | --- |
| Interleukin-2 | 1063 | 76.1 | White blood cell count | 24 | 1.7 |
| Interleukin-4 | 1063 | 76.1 | Neutrophil count | 24 | 1.7 |
| Interleukin-10 | 1063 | 76.1 | Hemoglobin | 24 | 1.7 |
| Tumor necrosis factor | 1063 | 76.1 | Platelet count | 24 | 1.7 |
| Interferon-γ | 1063 | 76.1 | Lymphocyte percentage | 24 | 1.7 |
| Interleukin-6 | 1042 | 74.6 | Lymphocyte count | 24 | 1.7 |
| CD4^+^ T cell percentage | 649 | 46.5 | Gender | 0 | 0 |
| CD8^+^ T cell percentage | 649 | 46.5 | Age(years) | 0 | 0 |
| CD4^+^/CD8^+^ ratio | 649 | 46.5 | Length of hospital stay | 0 | 0 |
| Immunoglobulin A | 612 | 43.8 | Use of intravenous immunoglobulin | 0 | 0 |
| Immunoglobulin G | 612 | 43.8 | Use of glucocorticoids | 0 | 0 |
| Immunoglobulin M | 612 | 43.8 | Use of antifungal agents | 0 | 0 |
| Erythrocyte sedimentation rate | 602 | 43.1 | Prior carbapenem exposure | 0 | 0 |
| D-dimer | 538 | 38.5 | Use of antibacterial drugs | 0 | 0 |
| Fibrinogen | 450 | 32.2 | Urinary catheterization | 0 | 0 |
| International standardized ratio | 450 | 32.2 | Indwelling gastric tube or drainage tube | 0 | 0 |
| Procalcitonin | 429 | 30.7 | Central venous catheterization | 0 | 0 |
| C-reactive protein | 42 | 3 | Mechanical ventilation | 0 | 0 |
| Neutrophil percentage | 24 | 1.7 | Invasive puncture | 0 | 0 |
| Red blood cell count | 24 | 1.7 | Flexible endoscopy | 0 | 0 |

# **Supplementary Table S2: LASSO-selected variables across five imputed datasets and the complete-case subset**

| **Variable** | **Imp 1** | **Imp 2** | **Imp 3** | **Imp 4** | **Imp 5** | **Selection Frequency** | **Complete case** |
| --- | --- | --- | --- | --- | --- | --- | --- |
| CD8^+^ T cell percentage | √ | √ | √ | √ | √ | 5 | √ |
| D-dimer | √ | √ | √ | √ | √ | 5 | √ |
| Fibrinogen | √ | √ | √ | √ | √ | 5 |  |
| Urinary catheterization | √ | √ | √ | √ | √ | 5 |  |
| Length of hospital stay | √ | √ | √ | √ | √ | 5 |  |
| Lymphocyte percentage | √ | √ | √ | √ | √ | 5 |  |
| Prior carbapenem exposure | √ | √ | √ | √ | √ | 5 | √ |
| Indwelling gastric tube or drainage tube | √ | √ | √ | √ | √ | 5 | √ |
| Mechanical ventilation | √ | √ | √ | √ | √ | 5 | √ |
| Gender | √ | √ | √ | √ | √ | 5 | √ |
| Use of antibacterial drugs | √ | √ | √ | √ |  | 4 |  |
| Use of antifungal agents | √ | √ | √ | √ |  | 4 |  |
| White blood cell count |  | √ | √ | √ | √ | 4 | √ |
| CD4^+^ T cell percentage | √ | √ |  | √ |  | 3 |  |
| Immunoglobulin M | √ | √ | √ |  |  | 3 | √ |
| Central venous catheterization |  | √ | √ | √ |  | 3 |  |
| Procalcitonin |  | √ | √ | √ |  | 3 |  |
| Platelet count |  | √ | √ | √ |  | 3 |  |
| Red blood cell count |  | √ | √ | √ |  | 3 |  |
| C-reactive protein |  | √ | √ |  |  | 2 |  |
| International standardized ratio |  | √ | √ |  |  | 2 |  |
| Use of intravenous immunoglobulin |  |  | √ | √ |  | 2 | √ |
| Immunoglobulin G |  | √ |  | √ |  | 2 |  |
| CD4^+^/CD8^+^ ratio |  | √ |  |  |  | 1 |  |
| Erythrocyte sedimentation rate |  |  | √ |  |  | 1 |  |
| Flexible endoscopy | √ |  |  |  |  | 1 |  |
| Hemoglobin |  |  | √ |  |  | 1 |  |
| Immunoglobulin A |  |  | √ |  |  | 1 | √ |

*Imp 1-5: the five multiply imputed datasets. Complete case: analysis performed on the subset of patients with no missing values (n = 304). √ indicates the variable was selected by LASSO at$\text{ }\text{λ}\text{.}\text{min}$. Only variables selected in at least one analysis are shown.*

**Supplementary Table S3: Performance of the logistic regression model on the training and internal test sets.**

| Metrics | Training Set | Test Set |
| --- | --- | --- |
| ROCAUC | 0.819 (0.783, 0.856) | 0.795 (0.729, 0.860) |
| PRAUC | 0.498 (0.398, 0.577) | 0.496 (0.373, 0.627) |
| Sensitivity | 0.740 (0.661, 0.807) | 0.727 (0.626, 0.825) |
| Specificity | 0.759 (0.730, 0.788) | 0.766 (0.723, 0.814) |
| Precision (PPV) | 0.365 (0.304, 0.414) | 0.366 (0.287, 0.450) |
| NPV | 0.940 (0.920, 0.958) | 0.938 (0.912, 0.962) |
| Brier Score | 0.103 (0.090, 0.116) | 0.104 (0.082, 0.123) |

**Supplementary Table S4: Odds ratios from the logistic regression model**

| Variable | OR (95% CI) | P value |
| --- | --- | --- |
| Mechanical ventilation | 3.424 (1.976, 6.050) | <0.001 |
| CD8^+^ T cell percentage | 1.059 (1.041, 1.078) | <0.001 |
| Urinary catheterization | 0.479 (0.287, 0.795) | 0.005 |
| Lymphocyte percentage | 0.982 (0.969, 0.995) | 0.006 |
| Length of hospital stay | 1.009 (1.003, 1.016) | 0.007 |
| Prior carbapenem exposure | 1.695 (1.087, 2.635) | 0.019 |
| Gender (female) | 1.322 (0.879, 1.979) | 0.177 |
| Fibrinogen | 0.896 (0.746, 1.071) | 0.233 |
| Indwelling gastric tube or drainage tube | 1.436 (0.758, 2.723) | 0.266 |
| D-dimer | 0.990 (0.960, 1.013) | 0.474 |

# Supplementary Figures


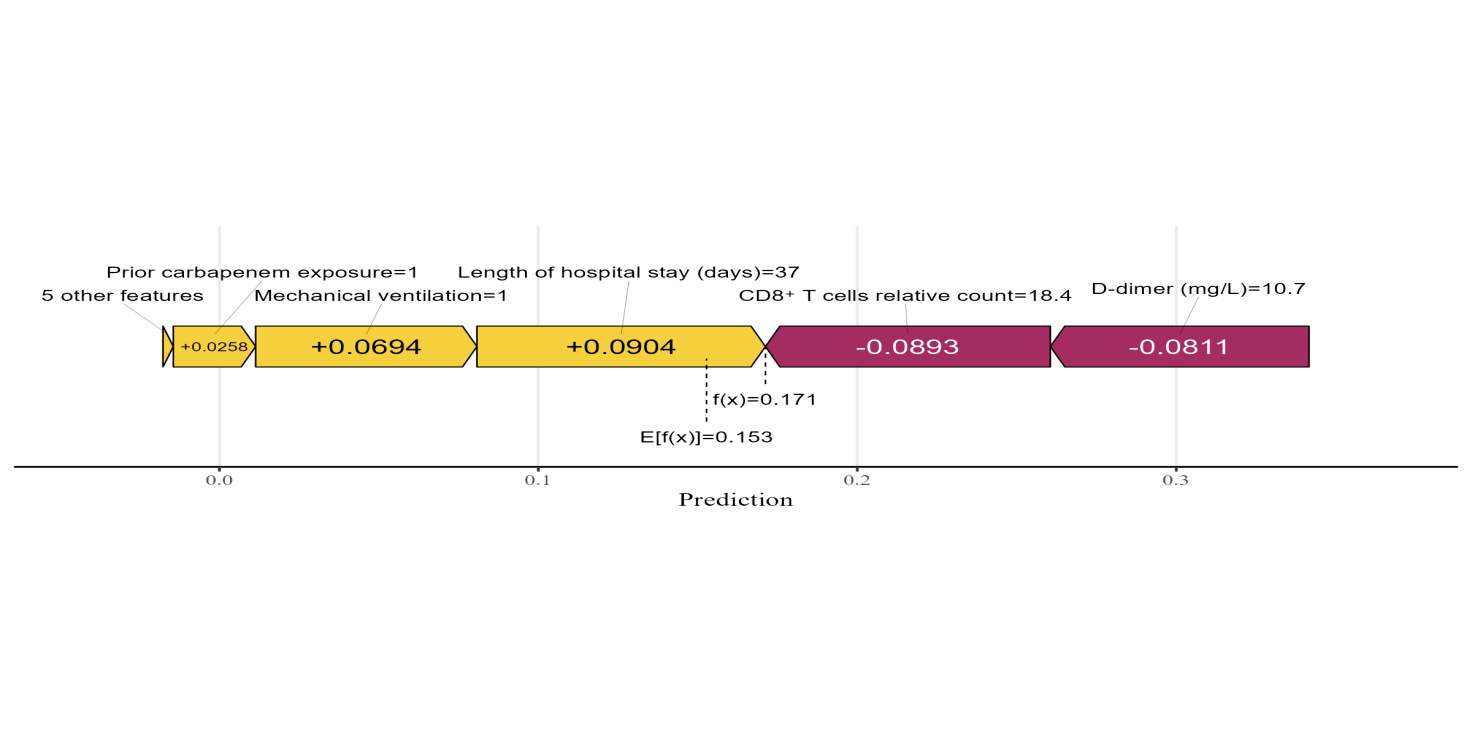


**Supplementary Figure S1.** Local Interpretation of CRPA Infection Risk Prediction Using the SHAP Method. The plot displays the SHAP values of key features contributing to the model's output for a single sample. Positive values (yellow) indicate risk-increasing factors, while negative values (purple) indicate risk-decreasing factors.
